# Role of linear endosonography in the diagnosis of biopsy‐negative malignant esophageal strictures: Exploring the unexplored

**DOI:** 10.1002/jgh3.12225

**Published:** 2019-07-18

**Authors:** Amol S Dahale, Siddharth Srivastava, Ujjwal Sonika, Ashok Dalal, Aditi Goyal, Puja Sakhuja, Sanjeev Sachdeva, Amarender S Puri

**Affiliations:** ^1^ Department of Gastroenterology G B Pant Institute of Postgraduate Medical Education and Research New Delhi India; ^2^ Department of Pathology G B Pant Institute of Postgraduate Medical Education and Research New Delhi India

**Keywords:** endoscopic ultrasound, esophageal stricture, malignancy

## Abstract

**Background and Aim:**

Endoscopic biopsy is standard for the diagnosis of esophageal malignancy. However, few cases present with smooth stricture with repetitive negative biopsy results. We aimed to use linear endoscopic ultrasound (EUS) and fine‐needle aspiration (FNA) in the diagnosis of biopsy‐negative suspected malignant esophageal strictures.

**Methods:**

We retrospectively analyzed the data from August 2017 to December 2018 of biopsy‐negative esophageal strictures. All adult patients with twice‐negative biopsies and with smooth overlying esophageal mucosa on endoscopy were included. Clinical, epidemiological, endoscopic, imaging, and EUS findings were noted and analyzed.

**Results:**

Eighteen patients underwent EUS for suspicion of malignant esophageal stricture. Seven were excluded as they were submucosal tumors. Eleven patients showed the presence of malignancy on EUS FNA samples. Nine were males. Computed tomography showed esophageal wall thickening in eight (16–38 mm) and esophageal mass in three patients. EUS showed loss of a normal five‐layered wall structure of the esophagus in all patients. Fine‐needle aspiration cytology demonstrated squamous cell carcinoma (*n* = 4), adenocarcinoma (*n* = 4), poorly differentiated carcinoma (*n* = 2), and neuroendocrine carcinoma (*n* = 1). There were no complications.

**Conclusion:**

EUS with FNA is effective and safe for the diagnosis of biopsy‐negative malignant esophageal strictures.

## Graphical abstract

Esophageal malignancy presenting as smooth esophageal stricture with a negative biopsy for malignancy is not uncommon. We retrospectively analyzed the data of 11 such patients who appeared to have malignant esophageal stricture otherwise but had negative repetitive biopsies. Linear endosonography provided a safe and effective modality for diagnosis in such patients.

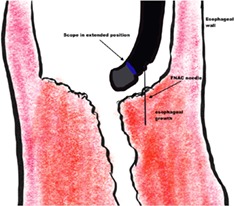

## Introduction

Endoscopic ultrasound (EUS) is a proven modality for the staging of esophageal cancer.^1^ However, its role in diagnosis is not well studied. Endoscopic biopsy remains the mainstay for the diagnosis of esophageal cancer.^2^ Esophageal cancer presenting as stricture with smooth overlying mucosa is uncommon but not rare.^3^ Endoscopic biopsy does not yield diagnosis in all such cases. Various alternative modalities have been tried in this scenario such as dilatation followed by biopsy or ultrathin endoscopy and biopsy.^4^ Dilation carries an inherent risk of perforation, and ultrathin endoscopy may not be possible in all the patients.^5^, ^6^ The yield of biopsies post dilation and post ultrathin endoscopy is also limited.^3^, ^7^ Nonetheless, definitive diagnosis is essential for the management of these patients, especially in view of advances in chemoradiotherapy.^8^, ^9^ Few case reports and small case series have shown the role of EUS with or without fine‐needle aspiration cytology (FNAC) in such patients.^3^, ^7^, ^10^ Here, we present our experience on the role of linear EUS in diagnosing esophageal cancer presenting as a smooth stricture.

## Methods

We retrospectively analyzed all patients with biopsy‐negative esophageal stricture at G B Pant Institute of Postgraduate Medical Education and Research, New Delhi, India from August 2017 to December 2018. Patient consent was waived by the institutional ethical committee due to the study's retrospective nature. Patients who were suspected to have malignant esophageal stricture but had negative biopsies were included in the study. Patients with incomplete records and those with submucosal tumors were excluded as the role of EUS is well established in submucosal tumors.

### *Patient evaluation*

All patients who presented to the outpatient department with dysphagia and a high suspicion of esophageal malignancy were evaluated using the standard protocol. Cross‐section imaging and esophagogastroscopy were performed in all the patients.

### *Endoscopic biopsy protocol*

Upper gastrointestinal endoscopy was performed with the Olympus GF‐H180 series forward‐viewing scope. Patients underwent endoscopy under local anesthesia using lignocaine 10% spray. As per our institutional protocol, endoscopic biopsies were taken using reusable biopsy forceps (Telemed Systems, Inc., Hudson, MA, USA). Four biopsy pieces were taken from the representative abnormal area. If this biopsy turned out to be negative for malignancy, a repeat biopsy with biopsy‐on‐biopsy (well biopsy) technique was taken, and a minimum of five pieces were obtained.

If the second biopsy was also negative for malignancy, then patients underwent ultrathin endoscopy. Patients with tight stricture, where even ultrathin endoscopy was not possible or ultrathin endoscopy was nonproductive, underwent linear EUS for evaluation and FNAC.

### *EUS protocol*

We used an Olympus GF‐UCT180 linear echoendoscope (Olympus Inc., Shinjuku, Japan). The procedure was explained to all patients, and informed consent was obtained. The procedure was performed under sedation using intravenous midazolam and propofol.

**Figure 1:**
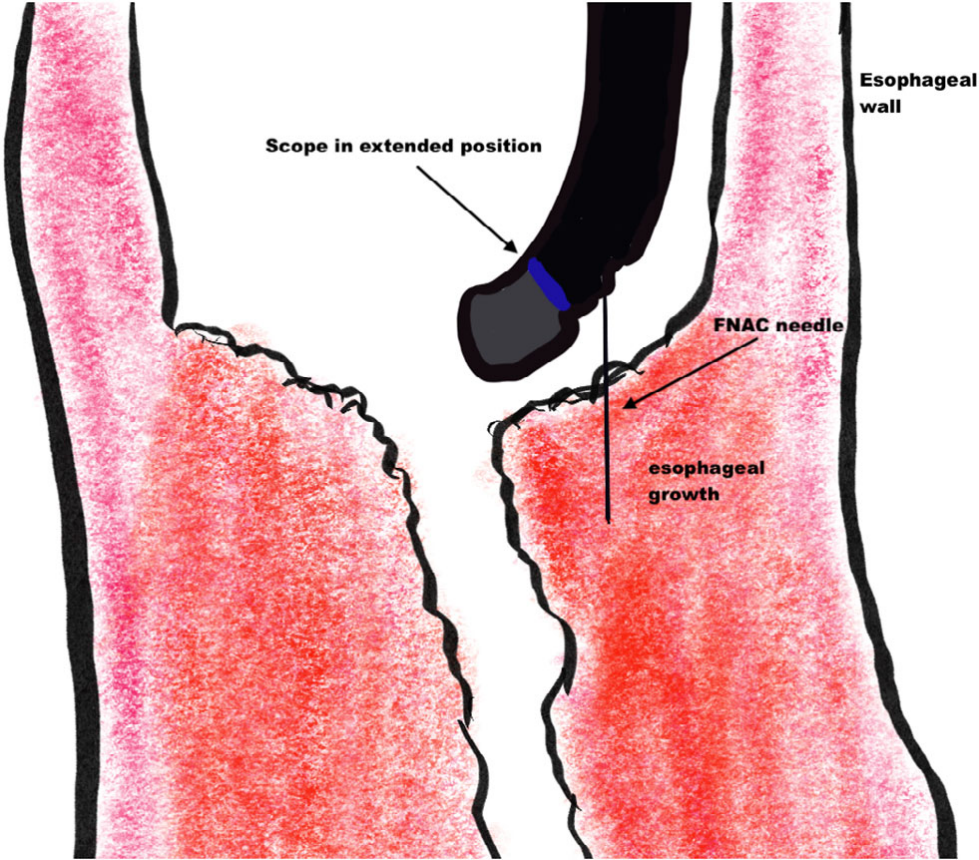
Graphical depiction of scope position in relation to stricture for evaluation and fine‐needle aspiration.

The EUS scope was inserted until 2 cm above the stricture under vision. The up–down knob of the EUS scope was placed in the down position, giving it an inverted hockey stick appearance (Fig. 1). The EUS mode was then activated. A gentle push was given to the scope enough to just kiss the stricture surface. With gentle up and down movements, we could obtain excellent views of the esophageal thickening or mass.

EUS‐fine‐needle aspiration (FNA) was performed for all patients. All FNA procedures were performed using a 22‐gauge FNA needle (EZ Shot 2, Olympus Inc.). Color flow and Doppler sonography were performed to avoid intervening vascular structures and to select a vessel‐free needle track. Once the tip of the sheath was visualized, the needle was advanced from the sheath through the wall under ultrasound guidance. The stylet was removed, and the initial passes were performed by moving the needle back and forth within the target lesion. No suction was applied during FNA unless the FNA did not yield any material. Three such passes were conducted, and an onsite pathologist was available to check the adequacy of the sample. Alcohol (95%)‐fixed slides and cell block were prepared immediately and sent for cytological and histological studies with HE and immunohistochemistry (IHC) if needed. All the patients were observed for 6 h postprocedure for any complications.

## Results

**Figure 2:**
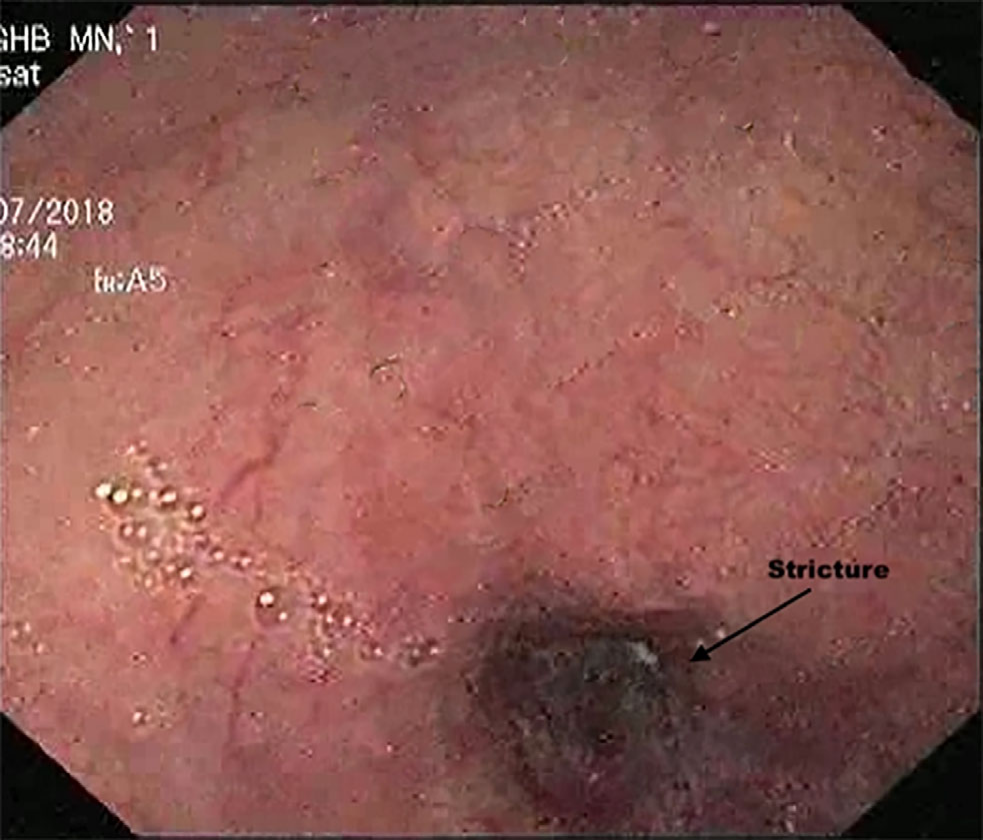
Endoscopic image showing esophageal stricture with smooth overlying mucosa.

**Figure 3:**
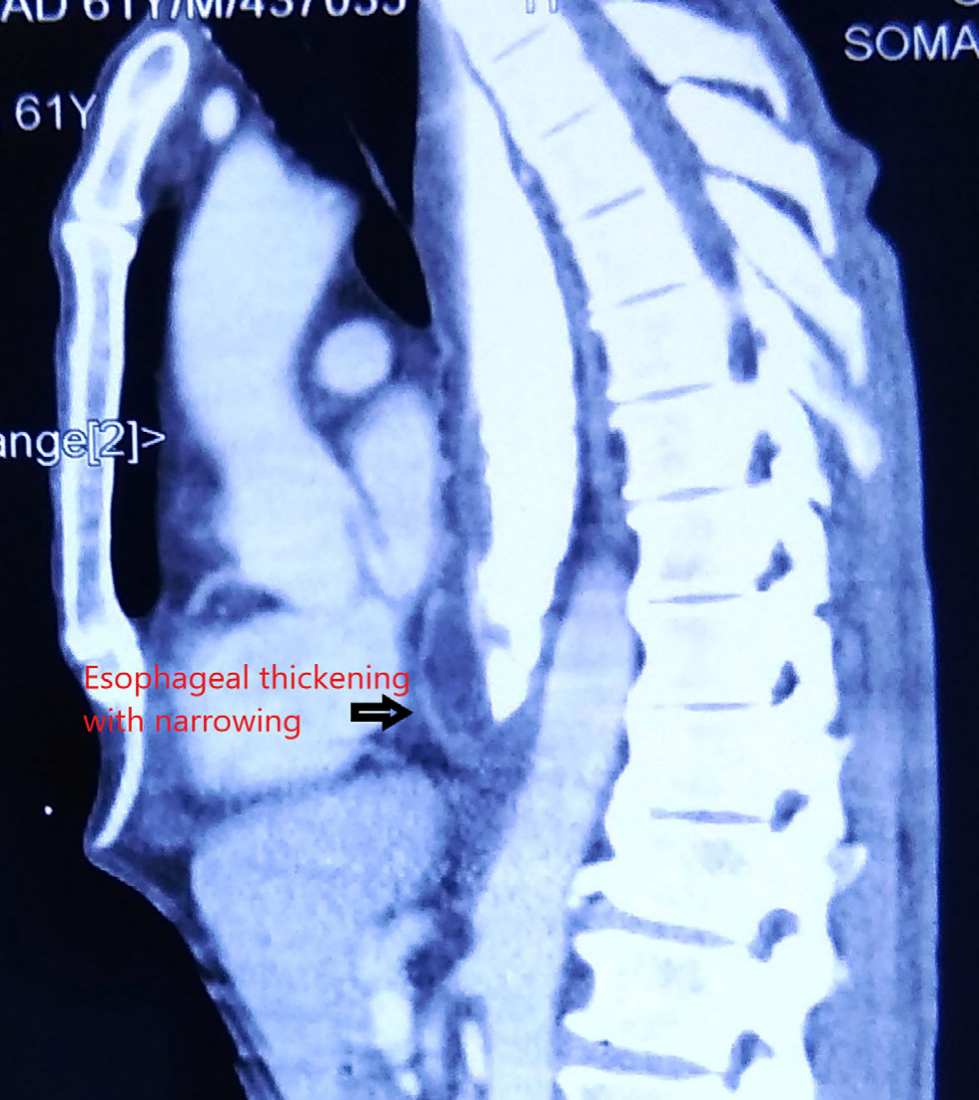
Computed tomographic image showing esophageal thickening with luminal narrowing.

A total of 123 patients underwent endoscopy and biopsy for suspected esophageal cancer during the study period, 23 of whom had an initial negative biopsy. Repeat biopsy was positive for malignancy in three more patients. Twenty patients were thus negative for malignancy even after repeat biopsy. On revaluation, all these patients had a smooth‐appearing stricture without obvious growth on endoscopy (Fig. 2), although cross‐sectional imaging depicted high suspicion of malignancy in view of asymmetrical thickening or mass (Fig. 3).

Of these 20 patients, only 4 could undergo successful ultrathin endoscopy and biopsy. However, of these four, only one was positive for malignancy, and the remaining three were still inconclusive. These 19 patients were planned for EUS. One patient was unable to undergo EUS due to poor ASA (American Society of Anaesthesiologists) physical status. Thus, a total of 18 patients underwent linear EUS examination with FNA. On cytological examination, seven were submucosal tumors, and 11 patients had esophageal malignancy. These 11 patients with esophageal cancer were included in the current analysis.

The mean age was 57.9 ± 9.2 years (median = 60 years, range = 42–70 years), and nine were male. All patients presented with dysphagia. Median symptom duration was 2 months (range 3 weeks to 4 months). All patients showed either asymmetric esophageal wall thickening (*n*‐8) or a mass with surrounding infiltration (*n*‐3) on contrast‐enhanced computed topography scans. Median esophageal wall thickening was 24 mm (range = 16–38 mm).

### *EUS findings*

**Figure 4:**
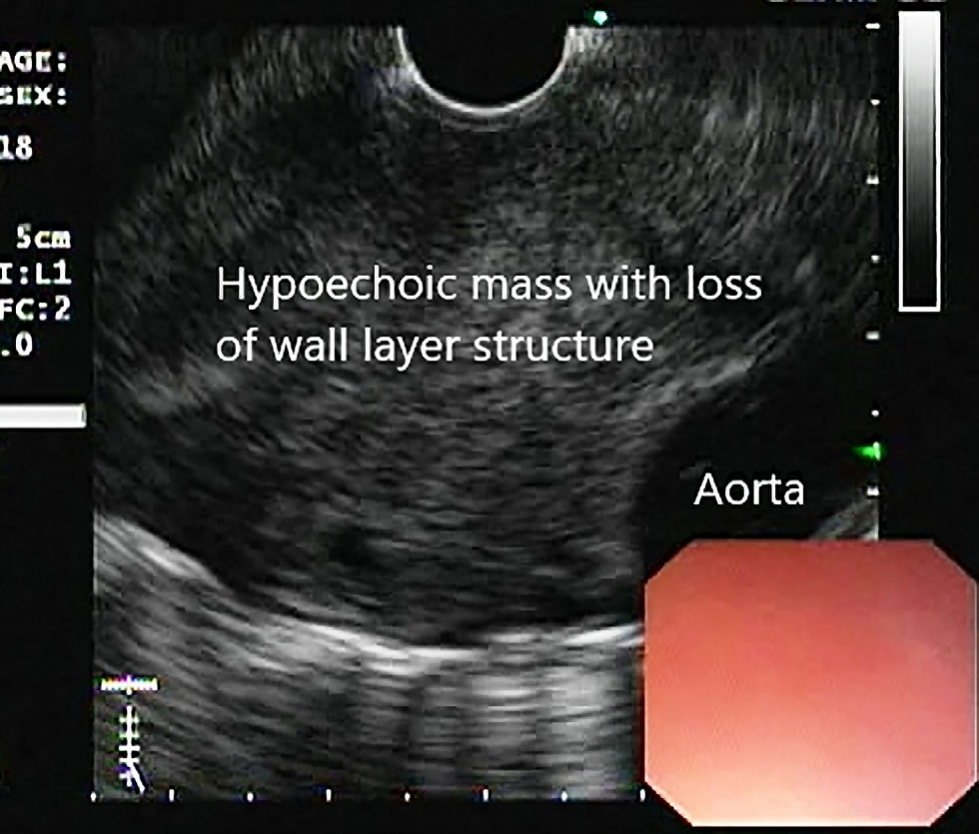
Linear endoscopic ultrasound image depicting mass with loss of wall layer structure of esophagus and mass abutting aorta.

EUS demonstrated hypoechoic wall thickening or mass lesion with loss of wall layer pattern in all the patients (*n* = 11) (Fig. 4). Obvious vascular involvement with loss of fat plane of the aorta was seen in five patients. Lymphadenopathy was observed in two patients. Both these cases had subcarinal lymph nodes. FNAC was performed on all patients with esophageal thickening or mass and additionally from lymph nodes in two patients. Tissue was taken both for slides and for cell blocks.

### *Pathology findings*

**Figure 5:**
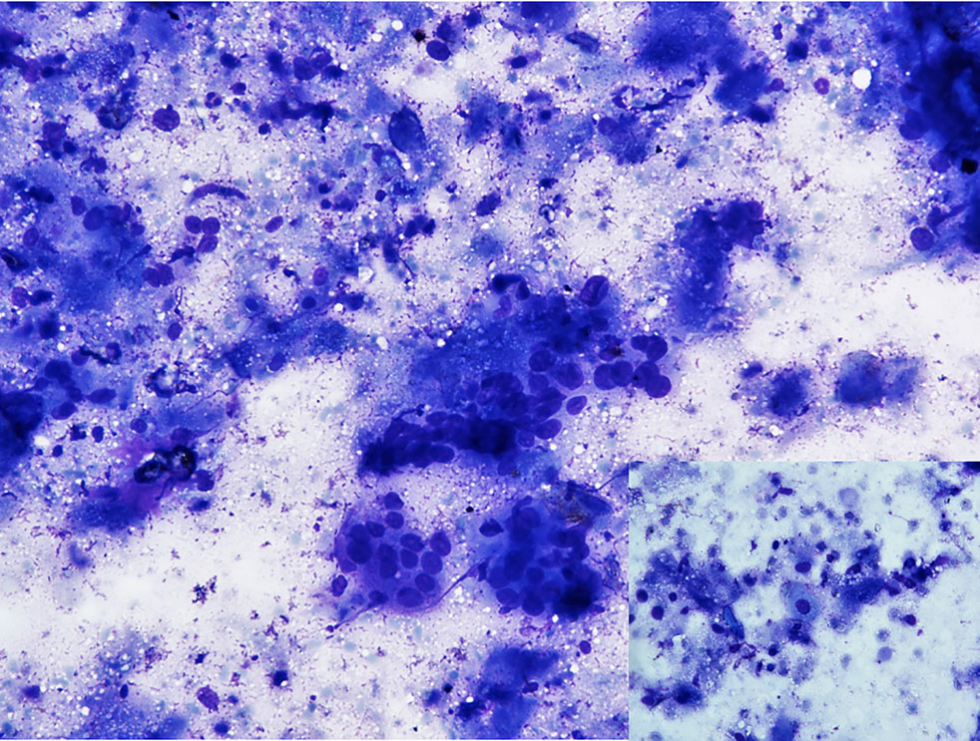
Giemsa‐stained smear showing clusters of atypical squamoid cells in a necrotic background (200×) in a case of squamous cell carcinoma esophagus. Inset shows squamous cell with dense blue cytoplasm.

Tissue smears and cell blocks demonstrated squamous cell carcinoma (SCC) (Fig. 5) in four (36%), adenocarcinoma in four (36%), and poorly differentiated carcinoma in two (18%) patients, while one patient had neuroendocrine carcinoma (9%). There were no complications related to procedure or sedation.

## Discussion

Our study shows the utility of linear EUS with FNA for the diagnosis of esophageal malignancy in patients with biopsy‐negative esophageal strictures**.** Endoscopy and biopsy has been the mainstay for the diagnosis of esophageal cancer, although biopsy yield is not always 100%.^2^ Smooth esophageal strictures, as in our series, are not a rare clinical problem. Apart from smooth strictures, verrucous variety, submucosal presentation of SCC, and secondary metastatic esophageal stricture may also produce biopsy negative results.^7^, ^10^, ^11^, ^12^, ^13^, ^14^

EUS has a well‐established role in the staging of esophageal cancer, but the role of EUS in its diagnosis is not well studied.^1^ The earliest report in biopsy‐negative malignant stricture is of radial EUS, and visual appearance was taken as the diagnostic criterion for malignancy, which was later confirmed on postoperative histopathological specimens.^3^ Considering the current advances in the management of esophageal malignancy, histopathological diagnosis is an earnest requirement. There is only one reported series of two cases in which patients underwent linear EUS and FNAC for esophageal strictures, but the EUS was performed after stricture dilatation.^7^ There is one study that described a large number (*n* = 23) of patients with esophageal or gastroesophageal junction malignancy, but all patients in this series presented with submucosal lesions, which were confirmed on EUS and EUS‐guided FNAC.^13^ Another case report from India described a patient with a smooth bulge in the esophagus that was diagnosed using EUS‐FNA, but the patient did not have esophageal stricture.^10^ What makes our study interesting is the presence of esophageal stricture, which makes EUS technically impossible, and the fact that we were able to perform EUS‐FNA without stricture dilatation. We have described a technique with subtle movements of the up–down knob following the placement of an EUS scope in an inverted hockey stick position, which provides both excellent echoendoscopic views and a good position for FNA. The EUS FNAC is a very safe technique with very few complications.^15^ Our study did not experience any complications. After establishment of diagnosis, three patients underwent curative chemoradiotherapy, two patients underwent surgical resection, and six patients underwent palliative chemoradiotherapy.

Our study has a few limitations, such as a small sample size and retrospective study design. Nonetheless, it is the first description of the role of linear EUS in this patient population. In our opinion, linear EUS with the described technique is safe and effective for obtaining tissue diagnosis in patients with biopsy‐negative malignant esophageal stricture.
